# Automated multi-subject fiber clustering of mouse brain using dominant sets

**DOI:** 10.3389/fninf.2014.00087

**Published:** 2015-01-12

**Authors:** Luca Dodero, Sebastiano Vascon, Vittorio Murino, Angelo Bifone, Alessandro Gozzi, Diego Sona

**Affiliations:** ^1^Pattern Analysis and Computer Vision Department (PAVIS), Istituto Italiano di TecnologiaGenova, Italy; ^2^Magnetic Resonance Imaging Department, Center for Neuroscience and Cognitive Systems@UniTn, Istituto Italiano di TecnologiaRovereto, Italy; ^3^NeuroInformatics Laboratory (NiLab), Fondazione Bruno KesslerTrento, Italy

**Keywords:** clustering, dominant sets, fibers segmentation, white matter, tractography, multi-subject, diffusion magnetic resonance imaging, DTI

## Abstract

Mapping of structural and functional connectivity may provide deeper understanding of brain function and disfunction. Diffusion Magnetic Resonance Imaging (DMRI) is a powerful technique to non-invasively delineate white matter (WM) tracts and to obtain a three-dimensional description of the structural architecture of the brain. However, DMRI tractography methods produce highly multi-dimensional datasets whose interpretation requires advanced analytical tools. Indeed, manual identification of specific neuroanatomical tracts based on prior anatomical knowledge is time-consuming and prone to operator-induced bias. Here we propose an automatic multi-subject fiber clustering method that enables retrieval of group-wise WM fiber bundles. In order to account for variance across subjects, we developed a multi-subject approach based on a method known as Dominant Sets algorithm, via an intra- and cross-subject clustering. The intra-subject step allows us to reduce the complexity of the raw tractography data, thus obtaining homogeneous neuroanatomically-plausible bundles in each diffusion space. The cross-subject step, characterized by a proper space-invariant metric in the original diffusion space, enables the identification of the same WM bundles across multiple subjects without any prior neuroanatomical knowledge. Quantitative analysis was conducted comparing our algorithm with spectral clustering and affinity propagation methods on synthetic dataset. We also performed qualitative analysis on mouse brain tractography retrieving significant WM structures. The approach serves the final goal of detecting WM bundles at a population level, thus paving the way to the study of the WM organization across groups.

## 1. Introduction

Diffusion magnetic resonance imaging (DMRI) permits non-invasive investigation of the white matter (WM) structure based on the diffusion profile of water molecules in the brain. This technique can be used to estimate the orientation of fibers at the voxel level, which can in turn be used by a number of tractography algorithms to build global fiber trajectories (Basser and Jones, 2002; Tournier et al., 2004). One of the advantage of DMRI over other methods is that it provides neuroscientists and neurosurgeons with the possibility to non-invasively identify fiber bundles, i.e., groups of fibers belonging to the same anatomical regions. These bundles represent major pathways in the overall physical connectivity of the brain. All diffusion-based MRI techniques (e.g., DTI, HARDY, Q-Ball) provide whole brain tractography datasets that are large (typically more than 100,000 fibers), complex and multi-dimensional, as well as artifact prone (e.g., crossing and broken fibers, low fractional anisotropy near the cortex, etc.) thus greatly complicating the description of large-scale WM structure and limiting the clinical impact of this approach. In most instances, the identification of relevant bundles is carried out via manual identification of regions of interest (ROIs) corresponding to the main known pathways (Mori et al., 2005; Wakana et al., 2007; Catani and de Schotten, 2008). However, this analysis is strongly affected by the prior knowledge used to identify the structures and very much prone to operator bias.

Methods for the automatic decomposition of whole brain tractography into fiber bundles could greatly help reduce complexity and bias associated with manual segmentation. For this reason, there is an urgent need for (semi)-automatic tools determining the bundles within and across subjects with little or no human intervention. This approach, frequently referred to as tractography segmentation, aims at generating a simplified representation of the WM structure, enabling easier navigation and improved understanding of the structural organization of the brain and its overall connectivity.

To automate bundles retrieval, various methods, based on different computational paradigms were proposed over the last few years. For example, the solution proposed in Li et al. (2010) is an evolution of the ROI-based technique that works directly on fiber and applies prior knowledge to perform preliminary parcellation of the brain. Kernel-PCA and C-means are then used to cluster the fibers. However, this approach is limited by the level of detail of the brain atlases, which can prevent the retrieval of small structures or suffer from cross-subject misalignments. Supervised methods were also proposed to retrieve local WM bundles using prior knowledge (Mayer et al., 2011; Olivetti and Avesani, 2011). These approaches require a first manual intervention to select tracts of interest in a subset of subjects and then retrieve the same structure in other subjects, and making them unsuitable for a global WM segmentation.

Clustering approaches represent a logical alternative to supervised methods as they permit to discover bundle structures without the need of prior anatomical knowledge. A common clustering framework is based on the exploitation of the affinity matrix of a single subject that indicates the similarity between each pair of fibers (Brun et al., 2004; O'Donnell et al., 2006; Zhang et al., 2008). A limitation common to all algorithms based on affinity matrix is their propensity to suffer from computational load owing to the calculation of pairwise distances between streamlines. Usually the complexity of these algorithms is 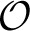
(*N*^2^), where *N* is the total number of fibers. Approaches to reduce computational complexity have been proposed like Quick Bundles (Garyfallidis et al., 2012), on line agglomerative clustering (Demir et al., 2013) and atlas-guided clustering with efficient implementation (Ros et al., 2013). A hierarchical clustering approach on single subject (Guevara et al., 2011) was proposed to automatically estimate the number of cluster from the dataset. However, the results of this approach are strongly conditioned by the number of hierarchical steps and several input parameters are required to carry out a comprehensive map of WM bundles.

Multi-subject spectral clustering (O'Donnell and Westin, 2007) was proposed to build a high dimensional WM atlas based on multiple DTI images. One limitation of this approach is that it needs prior information about the number of cluster to be segmented, which is however often unknown. To circumvent the problem of prior knowledge, multi-subject hierarchical clustering was proposed (Guevara et al., 2012). All subjects are registered to a common space but different manually agglomerative distance thresholds, based on neuroanatomical information, are used to retrieve the same WM bundles across different subjects.

A more advanced multi-subject clustering non-parametric Bayesian framework based on a Dirichlet process (Wang et al., 2011) was proposed to infer automatically the number of clusters from the data without affinity matrix computation. However, large datasets can dramatically decrease the quality of the results. Recently (Tunç et al., 2014) proposed a multi-subject adaptive clustering algorithm to build an atlas by using a subset of subjects to segment new subjects. However, manual thresholds are used to merge fibers and the atlas is strongly dependent of the number of subjects used.

In an attempt to circumvent all these limitations, we present a multi-subject clustering approach based on affinity matrices, directly connected with Graph Theory and rooted in the Game Theory. The method, based on the Dominant Set framework benefits from three properties that make it appealing for the problem at hand: (i) it is robust to noise and to outliers (Pavan and Pelillo, 2007); (ii) it is robust to parameters setting, generating stable results across different dataset (Dodero et al., 2013b); (iii) it automatically infers the number of clusters (Pavan and Pelillo, 2007). We tested our method on synthetic datasets comparing the results with state-of-the-art solutions like spectral clustering (Ng et al., 2002) and affinity propagation (Frey and Dueck, 2007). We also tested our method on a mouse brain dataset with the tractography inferred from DTI images, showing that it can reliably identify neuroanatomically plausible WM bundles in the mouse brain across multiple subjects without any prior neuroanatomical knowledge.

## 2. Materials and methods

**Figure 1:**
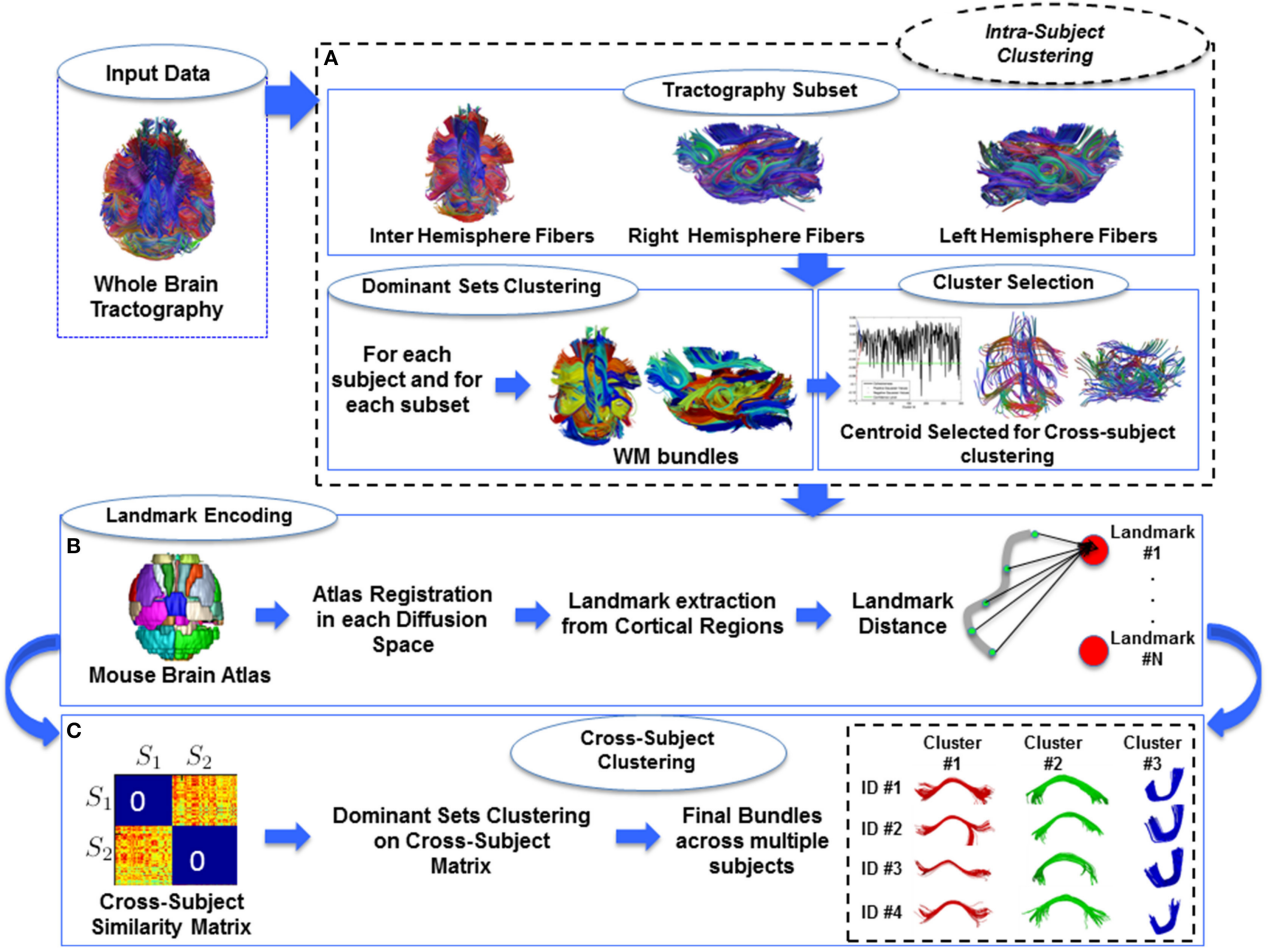
**Pipeline of our proposed method**. **(A)** Intra-subject clustering through Dominant Sets. **(B)** Landmark extraction and centroids encoding on the landmark space. Each centroids selected from intra-cluster step was encoded on landmarks. **(C)** Cross-Subject clustering through affinity block matrix and Dominant Set to find out same WM bundles across multi-subject.

Our main goal was to identify WM bundles across multiple subjects without prior registration of the raw diffusion data or the tractography. The algorithm approaches this problem in two steps. In the beginning, the tractography data-sets are segmented in the original diffusion space to obtain WM bundles for each subject, Dodero et al. (2013b). Subsequently, the bundles with high intra-subject similarity are clustered across subjects, performing all computations in the original space of the subjects by defining a space-invariant set of landmarks (O'Donnell et al., 2012). Figure 1 shows a schematic pipeline of the most important steps of our methods.

Since unsupervised learning methods can be heavily affected by the chosen similarity measure, and the two clustering levels use different metrics, we investigated and compared different measures, with the aim of finding the encoding that better preserves the relative similarities across metrics.

### 2.1. Standard fiber similarities

Each fiber is described by a sequence of points in 3D space. To achieve a uniform representation across fibers with the same number of equidistant points, each fiber was quantized using B-spline interpolation and sampling it with *k* = 12 points, as proposed in Garyfallidis et al. (2012). We thus coded the generic *i*-th streamline F_*i*_ as a 3D curve described by a constant sequence of points *F*_*i*_ = [**p**^*i*^_1_ … **p**^*i*^_*k*_] with **p**^*i*^_*j*_ ∈ ℝ^3^. Since fibers have no preferred orientation, also the flipped version of the streamlines *F*′_*i*_ = [**p**^*i*^_*k*_ … **p**^*i*^_1_] was considered in each metric computation.

To cluster WM at single-subject level, we compared the symmetrized mean closest point distance (Guevara et al., 2011) and symmetrized point to point distance.

- *Symmetrized mean closest point distance*
  (1) $$d_{smp}(F_{i},F_{j})=\frac{1}{2}\left(d_{m}(F_{i},F_{j})+d_{m}(F_{j},F_{i})\right)$$
  defined as the average of the two directed (non-symmetric) mean closest points distances between fibers *F*_*i*_ and *F*_*j*_.
  (2) $$d_{m}(F_{i},F_{j})=\frac{1}{k}\sum_{p_{k}^{i}\in F_{i}}\underset{p_{l}^{j}\in F_{j}}{\min}\|p_{k}^{i}-p_{l}^{j}{\|}_{2}$$
  where ‖ * ‖_2_ is the Euclidean norm.
- *Symmetrized Point to Point Distance*
  (3) $$d_{pp}(F_{i},F_{j})=\min\left(d_{p}(F_{i},F_{j}),d_{p}(F_{i},{F^{\prime }}_{j})\right)$$
  defined as the minimum of the two directed mean points distances between fibers *F*_*i*_ and *F*_*j*_ and its flipped version *F*′_*j*_.
  (4) $$d_{p}(F_{i},F_{j})=\frac{1}{k}\sum_{k}\|p_{k}^{i}-p_{k}^{j}{\|}_{2}$$
  where **p**^*i*^_*k*_ and **p**^*j*^_*k*_ are the corresponding points sampled in the two fibers.

Regardless the chosen metric, the affinity matrix *A* = *a*_*ij*_ encoding the fiber similarities was built:

(5) $$a_{ij}=\{\begin{array}{ll} e^{-\frac{d(F_{i},F_{j})}{\sigma }} & \text{ if }(i,j)\in E\\ 0 & \text{ otherwise}. \end{array}$$

where σ is a normalization term. We imposed σ = max_*i*,*j* (*d*(*F*_*i*_, *F*_*j*_))_ fixing a unique bound for *a*_*ij*_, regardless of the used dataset.

### 2.2. Landmark-based similarities

Starting from the brain atlas registered to each diffusion space, we define some landmarks (3D points in the volume), which have different spatial locations in each subject but refer to the same cortical structures across datasets. These points are used to represent the fibers with a cross-subject invariant descriptor, which allows us to avoid space registration, handling the fiber segmentation in the original space. More specifically, in our experiments with mice tractography landmarks were defined from an anatomical t2-weighted mouse brain atlas (Sforazzini et al., 2013) (139 brain regions) linearly registered to each subjects space, using FSL's FLIRT, v.5.0.6 (Smith et al., 2004). We next selected a subset of symmetric cortical and sub-cortical areas (50 labels), covering both hemispheres and including all the major cortical and subcortical districts of the mouse brain (Paxinos and Franklin, 2004), and for each ROI we computed the center of gravity obtaining fifty landmark points. We next tested two landmark-based measures, defined as follow:

- Symmetrized Minimum Landmark Distance
  Given the list of landmarks *L* = *L*_1_ … *L*_*n*_ (with *n* = 50 in our case) each one identifying a specific brain region, we built a corresponding feature vector $\tilde{F}$ (Dim. 1 × *n*) describing each fiber as the list of minimum distances between all the landmarks and the fiber itself. More specifically, each fiber was encoded as a vector $\tilde{F}$_*i*_ = {*f*^*i*^_1_, …, *f*^*i*^_*n*_} such that:
  (6) $$f_{s}^{i}=\underset{p_{k}^{i}\in F_{i}}{\min}\|p_{k}^{i}-L_{s}{\|}_{2}$$
  Then we define the similarity between fibers as:
  (7) $$d_{l}(F_{i},F_{j})=\;\|\tilde{F_{i}}-\tilde{F_{j}}{\|}_{2}$$
  and the affinity matrix was determined using Equation 5.
- Landmark Distance
  An alternative and more selective encoding can be obtained by employing a full landmark distance representation, where each point in a fiber is mapped using all elements in the landmark space. In this case each fiber is encoded into a vector $\tilde{F}$_*i*_ of dimensions *k* × *n*, where *k* is the number of sample points in a fiber and *n* is the number of landmarks and each entry of this vector is the Euclidean distance between one fiber's point coordinate and one landmark coordinate. We define $\tilde{F}$_*i*_ = {*f*^*i*^_11_, …, *f*^*i*^_*1n*_, …, *f*^*i*^_*k*1_, …, *f*^*i*^_*kn*_} such that:
  (8) $$f_{ks}^{i}=\;\|p_{k}^{i}-L_{s}{\|}_{2}$$
  Equation 7 was then used to compute the similarity and the corresponding affinity matrix was determined according Equation 5.

### 2.3. Metric comparison

**Figure 2:**
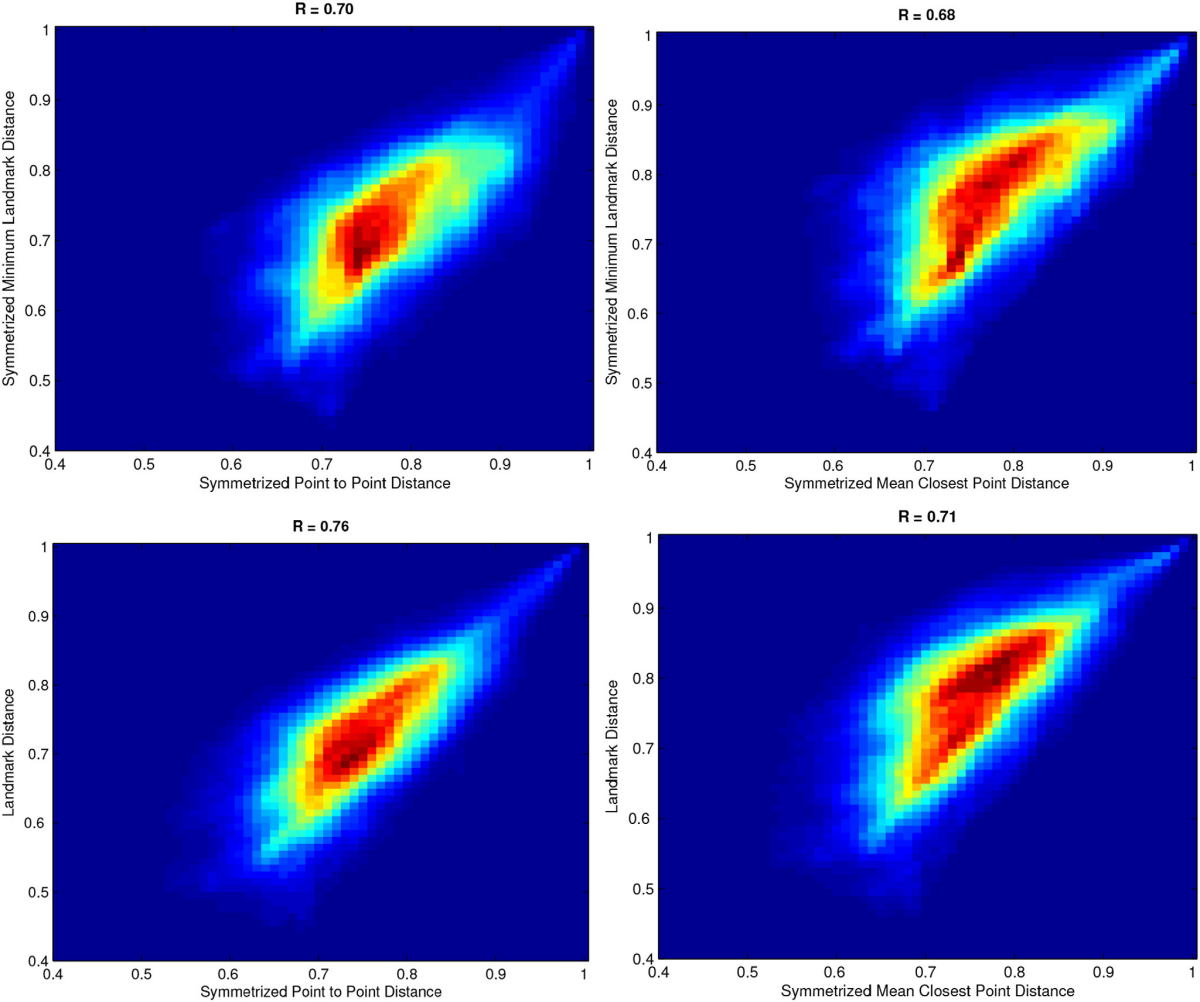
**2-D Histograms of affinity matrices using different similarity measure**. **Top-Left** symmetrized minimum landmark distance vs. symmetrized point to point distance. **Top-Right** symmetrized minimum landmark distance vs. symmetrized mean closest point distance. **Bottom-Left** landmark distance vs. symmetrized point to point distance. **Bottom-Right**: landmark distance vs. symmetrized mean closest point distance.

The above two groups of similarity measures were defined for the two clustering steps in the light of their different requirements. Since the choice of the similarity measure can greatly affect the clustering algorithms we compared the measures aiming at selecting the two that produce most similar results. The landmark measure is almost mandatory in order to avoid the tractography alignment. However, being the landmarks-based representation an approximation of the real fiber location, we have to choose the similarity between elements able to preserve the geometry and the shape of the subject bundles. We thus pairwise compared all proposed measures computing each similarity measure between each pair of fibers of a random subject. In Figure 2 are depicted the distributions of all pairwise comparisons. Comparing the the similarities with Pearson correlation we found that symmetrized point to point distance and landmark distance are the most correlated presenting the closest correspondence (see Figure 2-Bottom Left). Based on these results, we adopted the symmetrized point to point distance for intra-subject clustering and the landmark distance for cross-subject clustering.

### 2.4. Dominant sets clustering

Dominant Sets framework (Pavan and Pelillo, 2007) is a graph-theoretic method that generalizes the maximal clique problem to weighted graphs. It finds a compact, coherent and well-separated subset of nodes into a graph, i.e., the *dominant set* (DS). This framework defines the correspondence between clique, DS and cluster using a graph-theoretic perspective, and provides an optimization algorithm used to extract all DSs in a graph. Formally, a dataset is represented as a weighted undirected graph *G* = (*V*, *E*, ϕ) with no self-loop in which the vertices *V* are the data points and the edges *E* ⊆ *V* × *V* represent neighborhood relations among pairs of nodes, quantified by the weighting function ϕ : *E* → ℝ_+_. A DS formalizes two crucial properties of all clustering techniques: the *intra-cluster homogeneity* and *inter-cluster inhomogeneity*.

A graph is compactly represented by its weighted adjacency matrix *A* (the affinity matrix in our approach), which is defined by Equation 5. In our setting, each fiber corresponds to a node in the graph and the weighting function ϕ provides a measure of the similarity between pairs of fibers. Evaluating these two properties in all the possible subset of *V* is obviously unfeasible, for this reason the problem is casted into the following optimization task:

(9) $$\begin{array}{l} \text{maximize }x^{T}Ax\;\\ \text{subject to }x\in \;{△}^{n}\; \end{array}$$

where **x** lies in the standard n-dimensional simplex Δ^*n*^, or equivalently, ∑_*i*_ *x*_*i*_ = 1, ∀ *i x*_*i*_ ≥ 0. In the DS framework, **x** is called the *weighted characteristic vector* and it quantifies the degree of participation of the *i*-th component in the DS. If **x** is a strict local solution of (9) then its support, defined as δ(**x**) = {*i* | *x*_*i*_ > 0}, is a DS (Pavan and Pelillo, 2003) and thus a cluster. A local maximizer of (9) is found using the *replicator dynamics*(Pavan and Pelillo, 2003), a result from the evolutionary game theory mimicking the temporal changes in a population, based on the fitness of its individuals:

(10) $$x_{i}(t+1)=x_{i}(t)\frac{{\left(Ax(t)\right)}_{i}}{x{\left(t\right)}^{T}Ax(t)}$$

The optimization starts with a point **x**(*t*_0_), sited in the barycenter of the simplex $\left(x_{i}(t_{0})\;=\;\frac{1}{n},\forall i\right)$. Equation (10) is iterated until stability which is guaranteed to be reached if the matrix *A* is non-negative and symmetric. Theoretical stability condition is achieved when **x**(*t* + 1) = **x**(*t*), i.e., when the distance between two consecutive steps ‖**x**(*t* + 1) − **x**(*t*)‖ is lower than a threshold ϵ (in our setting ϵ = 10^−7^). Equation (10) also guarantees the satisfaction in time of constraint in Equation (9) (Pavan and Pelillo, 2003). In practice, the algorithm operates a selection process over the components of vector **x** driven by the affinity matrix *A*. At convergence some elements of **x** will emerge (*x*_*i*_ > 0) and others will become extinct (*x*_*i*_ = 0). In order to extract multiple clusters a *peeling-off* strategy is applied: once a DS is determined, it is removed from the whole set of vertices *V*, and the process is iterated on the remaining nodes, until all elements are clustered.

Applying the method in practical cases rarely produces a vector **x** whose certain elements are equal to zero and this is mainly due to the numerical approximation or premature stopping of the dynamics. Thresholding over **x** is thus integrated into the support calculation:

(11) $$\tilde{\delta }(x)=\{i|\;x_{i}>\theta *\max(x)\}\;\theta \in [0,1]$$

Small θs act as noise reducer, while higher values guarantee a greater number of clusters, each one having higher internal compactness. We fixed the coherence threshold according to the findings in a previous work (Dodero et al., 2013b), which needs to be very small to make the model stable (θ = 10^−5^).

### 2.5. Intra-subject clustering

**Figure 3:**
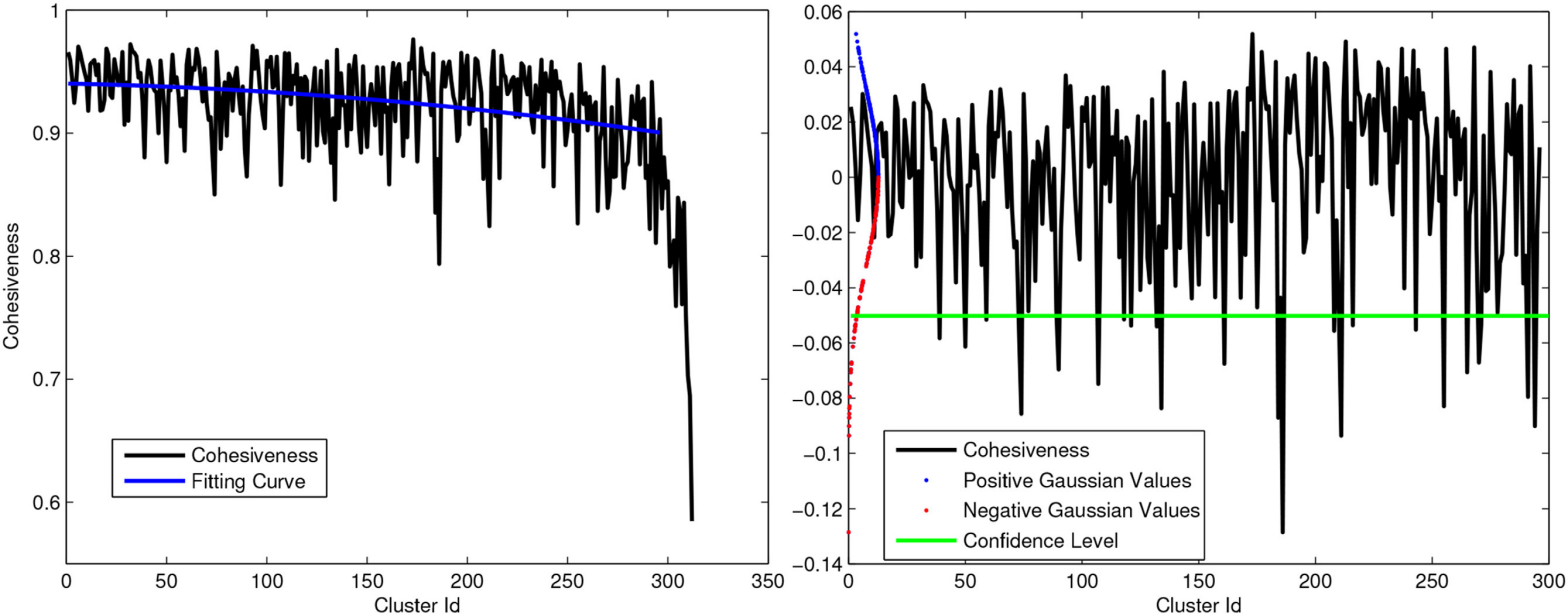
**Left:** Example of Cohesiveness curve and polynomial fitting. **Right:** Strategy to remove outliers from intra-subject clustering using gaussian curve and statical test. All positive peaks and negative above green line are considered as significant for multi-subject clustering.

DS clustering was first applied to single subject tractography volume to extract the WM bundles (intra-subject clustering). To reduce data dimensionality and thus computational complexity, we split the whole brain into three smaller datasets: left hemisphere, right hemisphere, and inter-hemispheric fibers, resulting in approximately 15,000 fibers per sub-datasets. The quality of retrieved bundles was then evaluated measuring the cohesiveness, which is a quantitative index measuring the internal coherence of each cluster δ as follows:

(12) $$C(\delta )=x^{T}Ax$$

where **x** is the characteristic vector corresponding to δ and *A* is the adjacency matrix. High values of cohesiveness are related to clusters with high internal similarity between elements while clusters with low cohesiveness aggregates fibers with little structural significance. Hence, we used the cohesiveness index to remove the less significant clusters. Figure 3-Left shows an example of cohesiveness determined for all iteratively generated clusters. Since the last generated clusters are generally not significant (Pavan and Pelillo, 2007), we removed the last 5% clusters which are mostly the cluster with very low internal cohesivity.

Moreover, in order to select most representative WM structures, we normalized the cohesiveness curve subtracting a second order polynomial curve fitted on the cohesiveness curve itself. Assuming the data distributed according to a Gaussian distribution 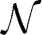
(0, σ), with σ estimated from the data, we decided to consider as outliers in term of cohesiveness all clusters in the negative tail of the distribution with a level of confidence *p* < 0.05. Figure 3-Right shows a plot of normalized coherence with the confidence level below which clusters are rejected. Once the set of cluster candidates were generated for each subject, the medoids were determined for each WM bundle and used as reference tracts in the next step.

### 2.6. Cross-subject clustering

In the proposed approach the bundles retrieved for all subjects separately were then clustered together in a second step according to the DS framework. To this purpose, clusters determined in the first step were substituted by their representative fiber (in our case the medoid) and then all dataset were joined into a single dataset in such a way that the algorithm groups bundles from different datasets while excluding pairs from the same dataset. In this way coherent clusters of bundles, including no more than one representative bundle from each dataset, were generated.

In more detail, given *n* datasets of bundles *D* = {*d*_1_, … *d*_*n*_} the extended dataset $\hat{D}$ obtained as the union of the elements in *D*, $\hat{D}$ = ∪^*n*^_*i* = 1_*d*_*i*_ is described by an affinity matrix. The graph based representation was then generated over $\hat{D}$ to avoid cliques containing bundles from the same subject. This was obtained by forcing the elements of the same subject to have zero similarity. The set of edges $\hat{E}$ in the graph describing the new dataset $\hat{D}$ is thus defined as:

(13) $$\hat{E}(i,j)=\{\begin{array}{ll} e^{-\frac{d(v_{i},v_{j})}{\sigma_{k,h}}} & \text{ if }v_{i}\in d_{k},v_{j}\in d_{h}\text{ and }k\neq h\\ 0 & \text{ otherwise}. \end{array}$$

where *v*_*i*_ and *v*_*j*_ are different elements in $\hat{D}$, *d*(·, ·) is a measure of distance between two elements, and σ_*k*,*h*_ is a normalization terms between datasets *h* and *k*. To obtain a metric *d*(·, ·) invariant to the different subject spaces, tracts where projected on the landmark space, and landmark distance was used to compare WM structures. The feature vector in the new space was determined according Equation 8 and a new similarity matrix was built. The resulting weighted adjacency matrix of $\hat{D}$ exhibits a “block shape” in which the main diagonal is composed of blocks of zeros ensuring that no pair of bundles from the same subject will appear in a cluster. Importantly, within this framework the algorithm can allow for and easily manage differences in the size of individual subject datasets.

**Figure 4:**
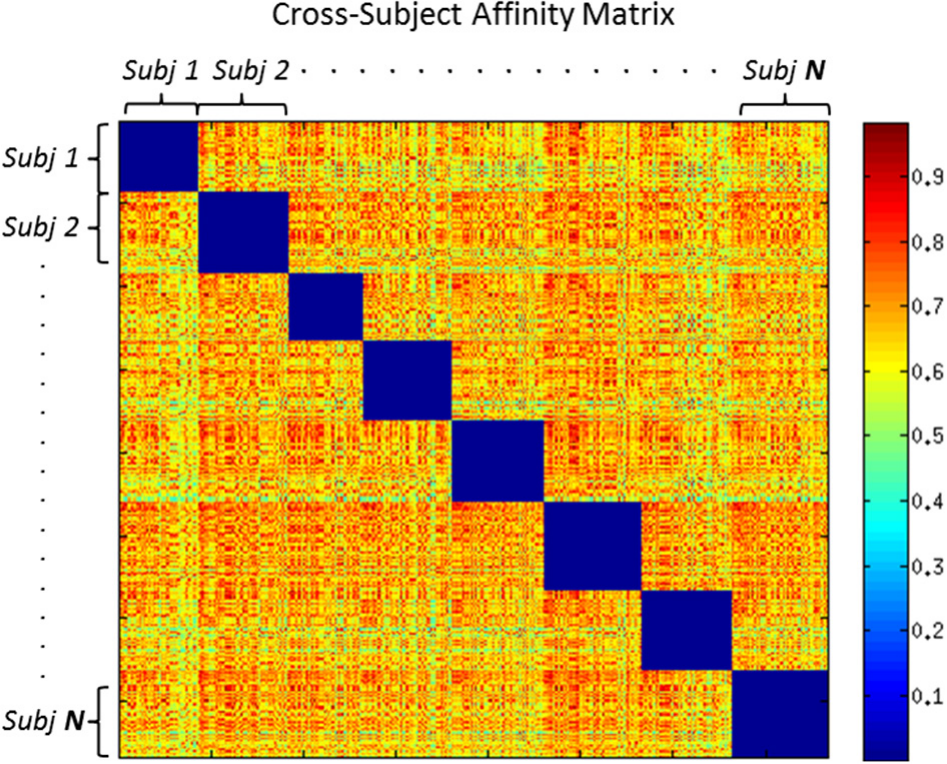
**Cross Subject Adjacency Matrix: each non-zero block represents the similarity between centroids across different subjects**.

Figure 4 shows an example of cross-subject affinity matrix, where the diagonal blocks represent the intra-subject similarity that we set to 0 to force a maximum of one bundle per subject in each cluster. The off-diagonal blocks describe the similarity between centroids of different subjects. We then applied DSs algorithm to the new adjacency finding similar WM bundles across multiple-subjects.

Aiming at finding the most important WM bundles we selected only significant bundles containing the maximum number of structures corresponding to the number of subjects. All clusters with fewer structures than the number of subjects were discarded, even if the internal cohesiveness was high. The analysis could in any case be further extended to other clusters that were currently rejected.

It can be proved that, if nodes *m*, *n* belong to the same dataset and their similarity is forced to be *a*_*mn*_ = 0 we are sure that the pair cannot be part of the same DS (cluster) and thus on each clusters we will have only the relationship between different datasets (the ones with positive weights).

### 2.7. Mouse brain dataset

All procedures were carried out in accordance with the European directive 86/609/EEC governing animal welfare and protection, which is acknowledged by the Italian Legislative Decree no. 116, 27 January 1992. The protocol was reviewed and consented to by the animal care committee of the Istituto Italiano di Tecnologia. All surgical procedures were performed under anesthesia.

DTI volumes from adult male 8 *ex vivo* wild type mouse brains (C57BL/6J, Charles River, Como Italy), an inbred strain widely used in neuroscience research, were acquired as previously described (Dodero et al., 2013a; Tucci et al., 2014). Briefly, sample preparation for *ex vivo* mouse brain imaging has been recently described in great detail (Dodero et al., 2013a; Tucci et al., 2014). Briefly, *ex vivo* high-resolution DTI images were acquired on paraformaldehyde fixed specimens and brains were imaged inside intact skulls to avoid post-extraction deformations. Diffusion tensor images (DTI) were acquired with 81 different gradient orientations at a *b*-value of 1262 s/mm^2^ (σ = 5 ms Δ = 10 ms), in-plane spatial resolution of 130 × 130 μm^2^, and slice thickness of 350 μm in the coronal plane, using a 4-shot EPI sequence with *TR* = 5500 ms and *TE* = 26 ms, 20 averages for a total acquisition time of 10 h 52 min. For each specimen, 8 co-centered volumes were acquired with no diffusion weighting (*b* = 0). Co-centered T2 weighted images were also acquired with the same resolution of the DTI volumes, using a 2-D fast spin-echo sequence.

Diffusion Tensor Tractography was performed by estimating the axonal fibers projections with the Fiber Assignment by Continuous Tracking (FACT) algorithm (Mori et al., 1999). Fractional Anisotropy (FA) threshold (0.1) and angle threshold (35°) were imposed to start and stop tracking. Fibers shorter than 3 mm were filtered out leading to a set of about 80,000 streamlines. Anatomical brain atlas of a C57BL/6J mouse brain (Sforazzini et al., 2013) was used to extract the landmarks needed for mapping the WM bundles cross-subjects. Homemade FA template was used to linearly register the mouse atlas in the subjects space.

### 2.8. Synthetic dataset

Synthetic WM streamlines and the associated DW-MR images were created using the numerical fibers generator software package (Close et al., 2009). The synthetic data has spherical volume with a fixed radius and composed of a random number of fibers and bundles. We used volumes released by the authors and 10 more volumes were generated in order to introduce more variability across dataset with an average of 41 ± 4 bundles and an 870 ± 37 fibers. Since the synthetic dataset does not contain group volumes, it was only used to compare our algorithm with the other state-of-the-art methods, i.e., spectral clustering and affinity propagation on the first step of the process, i.e., subject-wise fiber segmentation.

In particular, to perform a statistically robust comparison, for each of the above volumes we generated many trials randomly selecting a number of bundles with *k* = {5, 10, 15, 20, 25, 30}. This was repeated 5 time for each volume and for each cluster size. The empirical evaluation was therefore performed on a total of 510 random volumes with different number of clusters and fibers. We quantitatively evaluated the performance of all methods using some common indexes like completeness and adjusted rand index (Moberts et al., 2005).

## 3. Results

### 3.1. Clustering on synthetic datasets

For each method tested on synthetic dataset, we identified a set of optimal parameters. Spectral clustering requires a prior definition of the expected number of clusters *k*, which however is unknown in the address problem. Hence to avoid a biased evaluation, the algorithm was run with a varying number of clusters *k* ranging from 1 to 40 allowing a fair comparison. A similar requirement holds for both DS and affinity propagation. However, for both approaches empirical methods exist to decide proper parameter values required to obtain a number of clusters approximating the ground truth. Once optimal parameters are fixed, both DS and affinity propagation can then automatically find the optimal number of clusters.

More specifically, affinity propagation requires the definition of self-responsibility parameter, which according to the practice, if set *p* = min(*a*_*i*, *j*)_ is known to generate a number of cluster near the ground truth. DS framework instead requires fixing θ as described in Section 2.4. We used the Adjusted-Rand Index and Completeness indexes to evaluate the three methods, which are frequently used to evaluate the performance of clustering algorithms (Moberts et al., 2005). Higher completeness means that fibers belonging to the same anatomical bundle are clustered together. Rand index is defined as the number of agreement pairs divided by the total number of pairs. If the two partitions agree completely then the Rand index returns a value of 1, otherwise the lower-limit of this index is 0.

**Figure 5:**
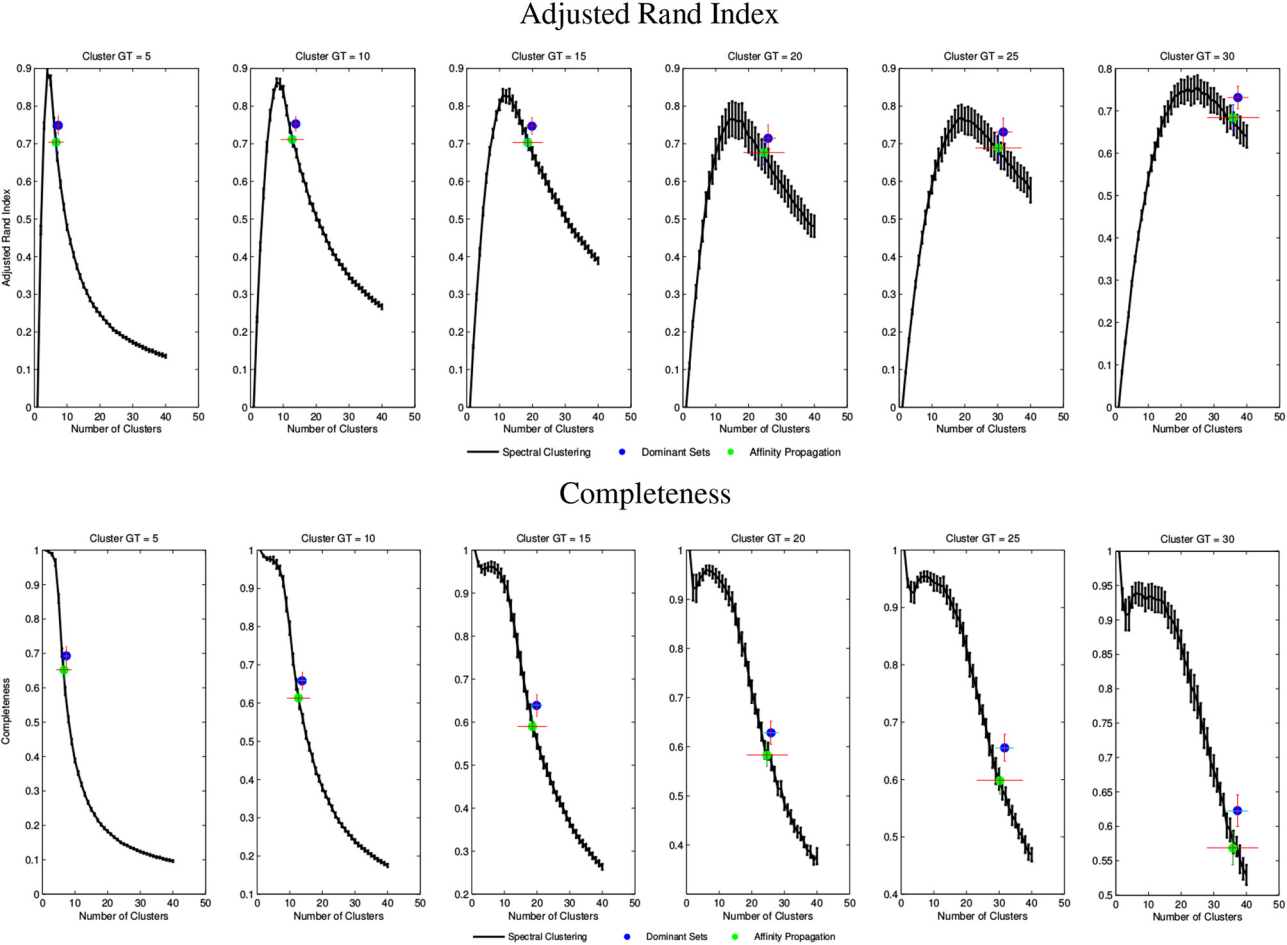
**Top Evaluation and comparison of DS through Adjusted Rand Index**. **Bottom:** Evaluation and comparison of DS through Completeness. Black curve shows Spectral Clustering performance and standard deviation. Blue and Green Dots show Dominant Set and Affinity Propagation performance. For all the boxes, x-axis represents the number of *k* clusters set for spectral clustering and retrieved for Dominant Sets and Affinity Propagation.

Figure 5 shows average results for spectral clustering, DSs, and affinity propagation with various dataset. The figure reports the results over the 6 groups of volumes, with varying amount of clusters {*k* = 5, 10, 15, 20, 25, 30}. DS algorithm always identifies a slightly greater number of clusters than the ground truth, an aspect that is not to be considered necessarily a drawback for the WM fiber segmentation. In general, DS and affinity propagation showed consistent output both in terms of number of cluster retrieved and quality of results. However, DS algorithm consistently showed higher completeness and adjusted rand index values. The results of spectral clustering also show that prior knowledge of the exact number of clusters could in principle produce higher performance (black curve). Affinity propagation exhibited similar performance than DSs although this approach suffers higher variance than DSs in term of number of clusters generated. DS, on the contrary, consistently yielded a solution approximating the optimal one and it was more reasonably stable across all experiments.

### 3.2. Clustering on real datasest

The proposed approach was also tested on a real dataset.

**Figure 6:**
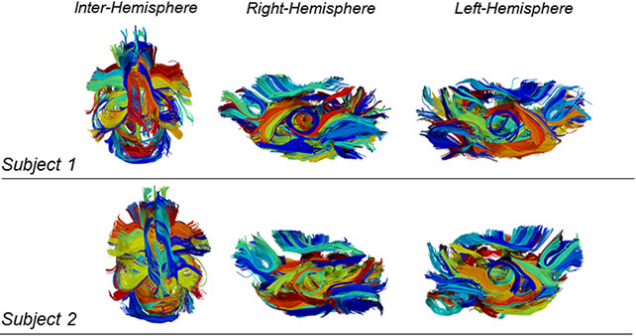
**Example of intra-subject clustering results on two mouse tractographies**. Each color is associated to a cluster of fibers. The two subjects have different color mappings because inter-subject clustering is not yet performed at this stage. While being the intra-subject clustering results different, there is a strong evidence of similarity in the determined structures.

Figure 6 shows two examples of qualitative results of intra-subject clustering applied to two mouse tractographies. We obtained different parcellation scheme for each subject and, at this level, each color does not represent associations between subjects.

**Figure 7:**
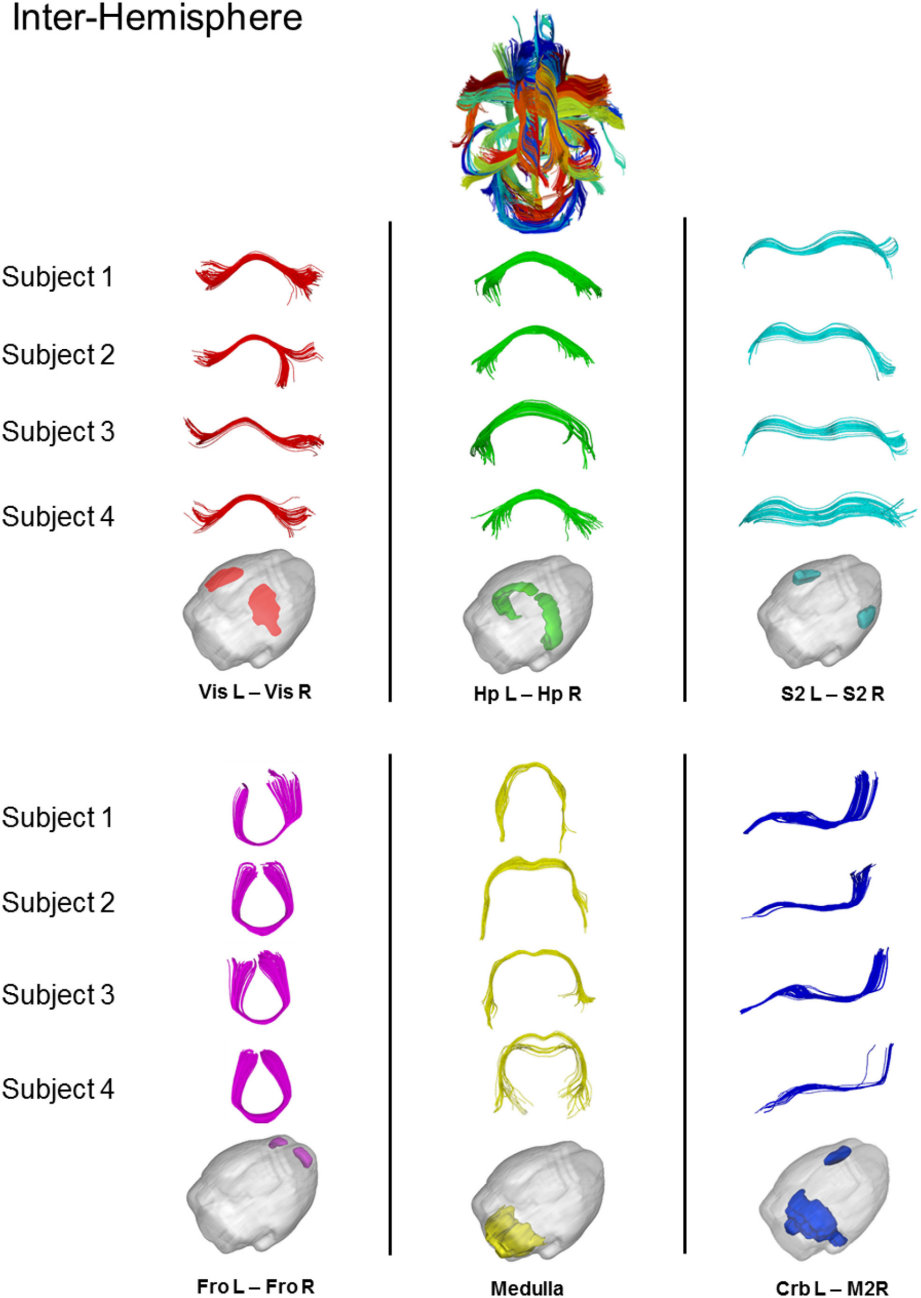
**Results of cross-subjects clustering on inter-hemispheric fibers and magnification of some significant white matter bundles**. For each significant bundles, we show four random subjects. Red = Dorsal Hippocampal Commissure, Green = Hippocampal Commissure, Cyan = Corpus Callosum, Magenta = Forceps Minor, Yellow = Posterio Commissure, Blue = Superior Rostro Caudal Tracts. Vis = Visual Cortex, Hp = Hyppocampus, S2 = Somato-Sensory Cortex, Fro = Cerebral cortex: frontal lobe, Crb = Cerebellum, M2 = Motor Cortex.

Figure 7 shows some examples of common inter-hemispheric WM bundles in 4 representative subjects (i.e., dorsal hippocampal commissure, hippocampal commissure, forceps minor, corpus callosum, and posterior commissure). Using the above restriction the algorithm was able to match 70 cross-subject bundles with significant inter-hemispheric commissure of multiple subjects clustered together. Despite the intrinsic variability of tractography across subjects, the algorithm automatically clustered bundles from different subjects.

**Figure 8:**
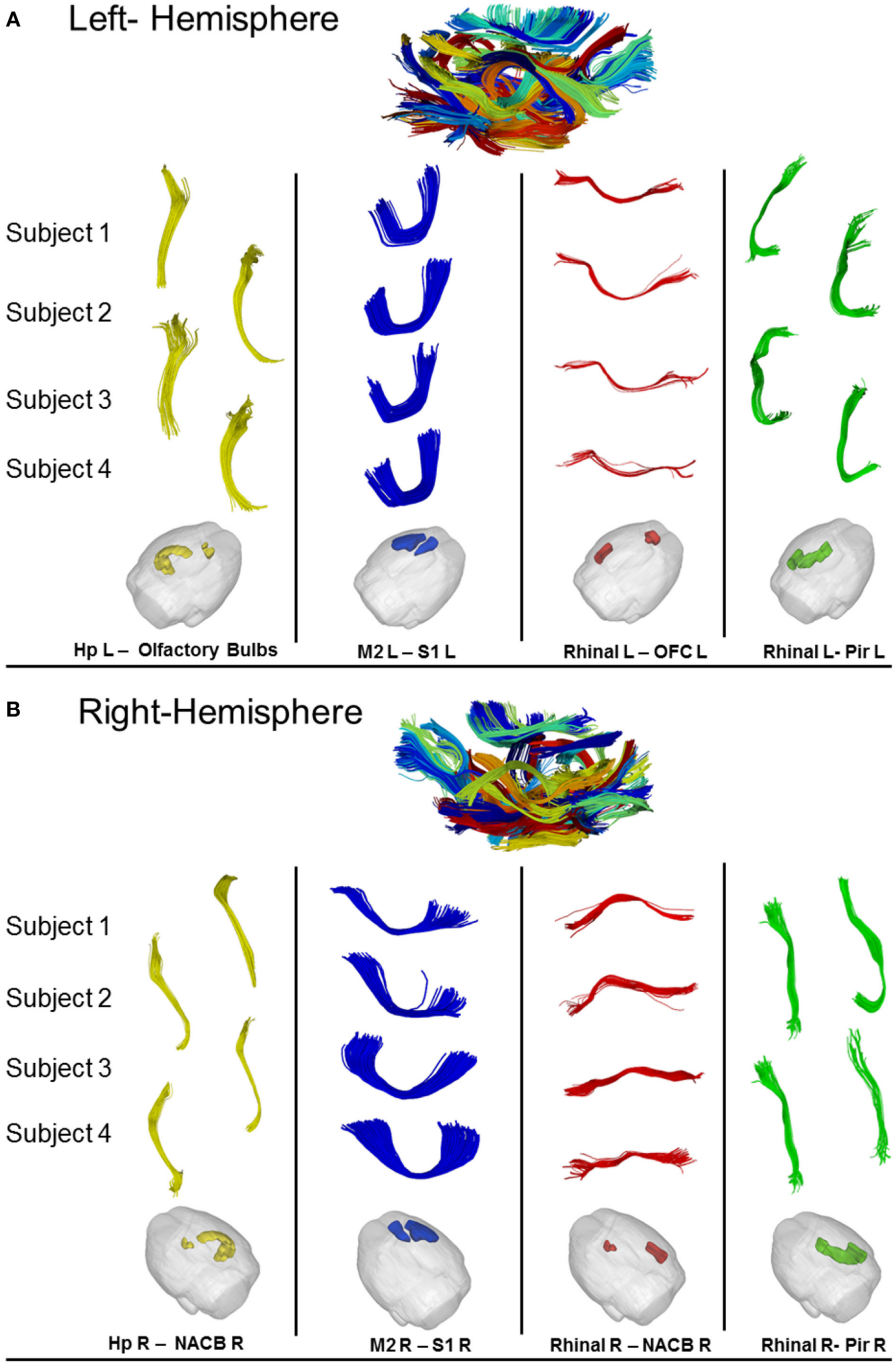
**(A)** Results of cross-subjects clustering on Left-Hemispheric fibers with some magnifications of relevant bundles. For each significant bundle we show four random subjects. **(B)** Results of cross-subjects clustering on Right-Hemispheric fibers with some magnification of relevant bundle. For each significant bundles we show four random subjects. Hp = Hyppocampus, S1 = Somato-Sensory Cortex, M2 = Motor Cortex, NACB = Nucleus Accumbens, Pir = Piriform Cortex, Rhinal = Rhinal Cortex, OFC = Orbitofrontal Cortex.

Figure 8 shows obtained results on left (A) and right (B) hemispheres, where the algorithm found, respectively 70 and 74 common WM bundles. Although no symmetry constraints were imposed, our method correctly identified inter-hemispheric bundles and preserved symmetry even in presence of different termination areas characterizing symmetric structures.

## 4. Conclusion and discussion

We presented a new method to cluster multiple-subject tractographies and to identify common bundles across subjects for the characterization of WM structure in a population. The proposed solution, based on DS can be used with diffusion MRI methods that use tractography to generate WM streamlines. We adopted DSs clustering to segment single subjects and we extended the framework to multiple subjects without resorting to spatial co-registration of the fibers, but using a landmark-based configuration.

Indeed, projection on the landmarks space, through linear registration of anatomical atlas on subject spaces, enables clustering of fibers in the original diffusion space, thus defining common structures across subjects while preserving invariance with respect to the intrinsic variability of each subject.

Clustering in the proposed multiple-subject framework requires different metrics to built affinity matrix for either the single or the cross-subject steps. Some similarity indexes in the space of streamlines were tested suggesting to use the symmetrized point to point distance (Equation 3) in the first stage and the landmark distance (Equation 8) in the second stage. We could have used the landmark projections for both steps, however, the symmetrized point to point distance is more robust in case of small fibers, while landmark distance, which is an approximation respect to the real distance between fibers, might fail in these cases. At single subject level is preferable to adopt a distance metric able to catch bundles characterizing the variability of each subject (O'Donnell and Westin, 2007; Guevara et al., 2011). On the other hand, the choice of landmark distance is mandatory to cast many subjects in a common space without registering the diffusion data.

We tested synthetic dataset for the proposed DS clustering and compared it with other methods, similarly working with adjacency matrix between fiber pairs, i.e., spectral clustering and affinity propagation. As mentioned in Section 2.4 we set θ very close to 0 according to our previous work (Dodero et al., 2013b). θ works as noise reducer and it acts on the internal elements of single cluster. With low values of θ we generally obtained low number of clusters but preserving high internal similarity. Conversely, higher values yielded over-segmentation, obtaining many clusters with just few elements. Moreover, adopting the fiber generator as ground truth and testing the performance of DSs, we obtained better values of completeness and adjusted rand index using θ very close to 0. From this indexes, we observed that our method is more suitable than the other two methods for fiber clustering. Indeed, unlike spectral clustering, our method does not need to set the number of clusters in advance, and is more stable than affinity propagation in terms of number of clusters generated. If the number of cluster is known a priori, spectral clustering works better than DS; however, the segmentation of whole tractography is an open problem where the number of WM bundles is typically unknown. In this framework, DS performs better compared to the other algorithms in a fair condition, i.e., with all algorithms generating the same number of clusters.

On real dataset, our algorithm was able to segment single subjects tractography generating anatomically plausible bundles. We did not observe any significant variation of WM bundles (also in the synthetic dataset) using various number of points to describe the fibers. We therefore used 12 points as suggested in Garyfallidis et al. (2012). According with DSs theory, the last clusters are always meaningless and they can be considered as outliers. Indeed, the choice to discard the last 5% of clusters is mostly empirical based on the data distribution.

In the cross-subject analysis, the number of landmarks has little influence on the matching between subjects. Indeed very few landmarks do not allow a proper representation of all fibers. On the other side to many landmarks while allowing a nearly perfect fiber representation induce an increased computational complexity. Our choice regarding the number of landmarks represent a good trade-off since they cover all the cortical brain regions, which represent the starting and end areas of the physical connections, while being still computationally manageable.

The algorithm was able to group coherent WM bundles of different subjects in their own space while preserving the symmetry of structures. Interestingly, this was obtained in presence of different shapes across subjects, demonstrating the robustness of the method. In principle, our approach enables the characterization of a population with significant bundles and could be applied to human data-sets to build an atlas of WM bundles for clinical applications.

### Conflict of interest statement

The authors declare that the research was conducted in the absence of any commercial or financial relationships that could be construed as a potential conflict of interest.

## References

[B1] Basser P. J. Jones D. K. (2002). Diffusion-tensor mri: theory, experimental design and data analysis–a technical review. NMR Biomed. 15, 456–467. 10.1002/nbm.783 12489095

[B2] Brun A. Knutsson H. Park H.-J. Shenton M. E. Westin C.-F. (2004). Clustering fiber traces using normalized cuts, in Medical Image Computing and Computer-Assisted Intervention–MICCAI 2004, eds Barillot C. Haynor D. R. Hellier P. (Saint-Malo: Springer), 368–375. 10.1007/b100265 PMC3296487 20209048

[B3] Catani M. de Schotten T. (2008). A diffusion tensor imaging tractography atlas for virtual *in vivo* dissections. Cortex 44, 1105–1132. 10.1016/j.cortex.2008.05.004 18619589

[B4] Close T. G. Tournier J.-D. Calamante F. Johnston L. A. Mareels I. Connelly A. (2009). A software tool to generate simulated white matter structures for the assessment of fibre-tracking algorithms. Neuroimage 47, 1288–1300. 10.1016/j.neuroimage.2009.03.077 19361565

[B5] Demir A. Mohamed A. Cetingul H. E. (2013). Online agglomerative hierarchical clustering of neural fiber tracts, in 2013 35th Annual International Conference of the IEEE Engineering in Medicine and Biology Society (EMBC) (Osaka: IEEE), 85–88. 10.1109/EMBC.2013.6609443 24109630

[B6] Dodero L. Damiano M. Galbusera A. Bifone A. Tsaftsaris S. A. Scattoni M. L.. (2013a). Neuroimaging evidence of major morpho-anatomical and functional abnormalities in the btbr t+tf/j mouse model of autism. PLoS ONE 8:e76655. 10.1371/journal.pone.0076655 24146902 PMC3797833

[B7] Dodero L. Vascon S. Giancardo L. Gozzi A. Sona D. Murino V. (2013b). Automatic white matter fiber clustering using dominant sets, in 2013 International Workshop on Pattern Recognition in Neuroimaging (PRNI) (Philadelphia, PA: IEEE), 216–219.

[B8] Frey B. J. Dueck D. (2007). Clustering by passing messages between data points. Science 315, 972–976. 10.1126/science.1136800 17218491

[B9] Garyfallidis E. Brett M. Correia M. M. Williams G. B. Nimmo-Smith I. (2012). Quickbundles, a method for tractography simplification. Front. Neurosci. 6:175. 10.3389/fnins.2012.00175 23248578 PMC3518823

[B10] Guevara P. Poupon C. Rivière D. (2011). Robust clustering of massive tractography datasets. Neuroimage 54, 1975–1993. 10.1016/j.neuroimage.2010.10.028 20965259

[B11] Guevara P. Duclap D. Poupon C. Marrakchi-Kacem L. Fillard P. Lebihan D.. (2012). Automatic fiber bundle segmentation in massive tractography datasets using a multi-subject bundle atlas. Neuroimage 61, 1083–1099. 10.1016/j.neuroimage.2012.02.071 22414992

[B12] Li H. Xue Z. Guo L. Liu T. Hunter J. Wong S. T. (2010). A hybrid approach to automatic clustering of white matter fibers. Neuroimage 49, 1249–1258. 10.1016/j.neuroimage.2009.08.017 19683061

[B13] Mayer A. Zimmerman-Moreno G. Shadmi R. Batikoff A. Greenspan H. (2011). A supervised framework for the registration and segmentation of white matter fiber tracts. IEEE Trans. Med. Imaging 30, 131–145. 10.1109/TMI.2010.2067222 20716499

[B14] Moberts B. Vilanova A. van Wijk J. J. (2005). Evaluation of fiber clustering methods for diffusion tensor imaging, in Visualization, 2005. VIS 05. IEEE (Minneapolis: IEEE), 65–72.

[B15] Mori S. Crain B. J. Chacko V. Van Zijl P. (1999). Three-dimensional tracking of axonal projections in the brain by magnetic resonance imaging. Ann. Neurol. 45, 265–269. 10.1002/1531-8249(199902)45:2<265::AID-ANA21>3.0.CO;2-3 9989633

[B16] Mori S. Wakana S. Van Zijl P. C. Nagae-Poetscher L. (2005). MRI atlas of human white matter. Am. Soc. Neuroradiol. 27, 1384–1385 Available online at: http://www.ajnr.org/content/27/6/1384.2.short

[B17] Ng A. Y. Jordan M. I. Weiss Y. (2002). On spectral clustering: analysis and an algorithm. Adv. Neural Inf. Process. Syst. 2, 849–856.

[B18] O'Donnell L. J. Westin C.-F. (2007). Automatic tractography segmentation using a high-dimensional white matter atlas. IEEE Trans. Med. Imaging 26, 1562–1575. 10.1109/TMI.2007.906785 18041271

[B19] O'Donnell L. Kubicki M. Shenton M. E. (2006). A method for clustering white matter fiber tracts. Am. J. Neuroradiol. 27, 1032–1036. Available online at: http://www.ajnr.org/content/27/5/1032.abstract 16687538 PMC2768142

[B20] O'Donnell L. J. Rigolo L. Norton I. Wells W. M. III. Westin C.-F. Golby A. J. (2012). fmri-dti modeling via landmark distance atlases for prediction and detection of fiber tracts. Neuroimage 60, 456–470. 10.1016/j.neuroimage.2011.11.014 22155376 PMC3423975

[B21] Olivetti E. Avesani P. (2011). Supervised segmentation of fiber tracts, in Similarity-Based Pattern Recognition, eds Pelillo M. Hancock E. R. (Venice: Springer), 261–274.

[B22] Pavan M. Pelillo M. (2003). A new graph-theoretic approach to clustering and segmentation, in Proceedings Computer Society Conference on Computer Vision and Pattern Recognition, Vol. 1 (Toronto: IEEE), I–145.

[B23] Pavan M. Pelillo M. (2007). Dominant sets and pairwise clustering. IEEE Trans. Pattern Anal. Mach. Intell. 29, 167–172. 10.1109/TPAMI.2007.250608 17108392

[B24] Paxinos G. Franklin K. B. (2004). The Mouse Brain in Stereotaxic Coordinates. Houston, TX: Gulf Professional Publishing.

[B25] Ros C. Güllmar D. Stenzel M. Mentzel H.-J. Reichenbach J. R. (2013). Atlas-guided cluster analysis of large tractography datasets. PLoS ONE 8:e83847. 10.1371/journal.pone.0083847 24386292 PMC3875498

[B26] Sforazzini F. Schwarz A. J. Galbusera A. Bifone A. Gozzi A. (2013). Distributed bold and cbv-weighted resting-state networks in the mouse brain. Neuroimage 87, 403–415. 10.1016/j.neuroimage.2013.09.050 24080504

[B27] Smith S. M. Jenkinson M. Woolrich M. W. Beckmann C. F. Behrens T. E. J. Johansen-Berg H.. (2004). Advances in functional and structural mr image analysis and implementation as fsl. Neuroimage 23 Suppl. 1:S208–S219. 10.1016/j.neuroimage.2004.07.051 15501092

[B28] Tournier J. Calamante F. Gadian D. G. Connelly A. (2004). Direct estimation of the fiber orientation density function from diffusion-weighted mri data using spherical deconvolution. Neuroimage 23, 1176–1185. 10.1016/j.neuroimage.2004.07.037 15528117

[B29] Tucci V. Kleefstra T. Hardy A. Heise I. Maggi S. Willemsen M. H.. (2014). Dominant β-catenin mutations cause intellectual disability with recognizable syndromic features. J. Clin. Invest. 124, 1468–1482. 10.1172/JCI70372 24614104 PMC3973091

[B30] Tunç B. Parker W. A. Ingalhalikar M. Verma R. (2014). Automated tract extraction via atlas based adaptive clustering. Neuroimage 102, 596–607. 10.1016/j.neuroimage.2014.08.021 25134977 PMC4252913

[B31] Wakana S. Caprihan A. Panzenboeck M. M. Fallon J. H. Perry M. Gollub R. L.. (2007). Reproducibility of quantitative tractography methods applied to cerebral white matter. Neuroimage 36, 630–644. 10.1016/j.neuroimage.2007.02.049 17481925 PMC2350213

[B32] Wang X. Grimson W. E. L. Westin C.-F. (2011). Tractography segmentation using a hierarchical dirichlet processes mixture model. Neuroimage 54, 290–302. 10.1016/j.neuroimage.2010.07.050 20678578 PMC2962770

[B33] Zhang S. Correia S. Laidlaw D. H. (2008). Identifying white-matter fiber bundles in dti data using an automated proximity-based fiber-clustering method. IEEE Trans. Vis. Comput. Graph. 14, 1044–1053. 10.1109/TVCG.2008.52 18599916 PMC2757786

